# Integral Characterization of Defective BDNF/TrkB Signalling in Neurological and Psychiatric Disorders Leads the Way to New Therapies

**DOI:** 10.3390/ijms18020268

**Published:** 2017-01-28

**Authors:** Gonzalo S. Tejeda, Margarita Díaz-Guerra

**Affiliations:** Instituto de Investigaciones Biomédicas “Alberto Sols”, Consejo Superior de Investigaciones Científicas-Universidad Autónoma de Madrid (CSIC-UAM), Arturo Duperier 4, 28029 Madrid, Spain; Gonzalo.Tejeda@glasgow.ac.uk

**Keywords:** BDNF, TrkB, excitotoxicity, stroke, neurodegeneration, neuroprotection, therapy

## Abstract

Enhancement of brain-derived neurotrophic factor (BDNF) signalling has great potential in therapy for neurological and psychiatric disorders. This neurotrophin not only attenuates cell death but also promotes neuronal plasticity and function. However, an important challenge to this approach is the persistence of aberrant neurotrophic signalling due to a defective function of the BDNF high-affinity receptor, tropomyosin-related kinase B (TrkB), or downstream effectors. Such changes have been already described in several disorders, but their importance as pathological mechanisms has been frequently underestimated. This review highlights the relevance of an integrative characterization of aberrant BDNF/TrkB pathways for the rational design of therapies that by combining BDNF and TrkB targets could efficiently promote neurotrophic signalling.

## 1. Introduction

Neurological disorders ranging from epilepsy to Alzheimer’s disease (AD), from stroke to Parkinson’s disease (PD), are currently estimated to affect as many as a hundreds of millions of people worldwide, and the number is expected to increase considerably in years to come. Stroke alone causes more than six million deaths per year, accounting for close to 11% of total world deaths. More than 47.5 million people are globally affected with dementia, with AD being the most common cause, while more than 50 million people suffer from epilepsy (WHO, 2016; [http://www.who.int/features/qa/55/en/]). Neuroprotective strategies have been developed to ameliorate brain damage by preservation or restoration of neurological functions. An extensively studied therapeutic strategy for the treatment of several brain diseases has been the administration of brain-derived neurotrophic factor (BDNF). This growth factor is central to the differentiation, maturation and survival of neurons. Despite its relative success in the laboratory, administration of neurotrophins did not produce the expected results in clinical trials. Thus, intrathecally administered BDNF in patients of amyotrophic lateral sclerosis (ALS) did not show significant effects on motor function and survival [1] or autonomic nervous system function [2]. These failures have been attributed to the multimodal nature of disease progression, poor neurotrophin delivery to appropriate targets due to limited blood–brain barrier (BBB) permeability and tissue diffusion, short serum half-lives and side effects [3]. In order to overcome these limitations, new molecules that mimic BDNF functions are currently under development. However, most neurological disorders not only show a dysregulation of BDNF but also an impairment of its downstream effectors whose relevance as pathological mechanisms needs to be valued. This review tries to integrally consider and understand the underlying mechanisms that affect each level of the BDNF signalling pathway. This knowledge represents an opportunity for a guided design of viable and efficient therapeutic tools to treat brain diseases.

## 2. Physiological Function of BDNF/TrkB Signalling in the Nervous System

**Figure 1:**
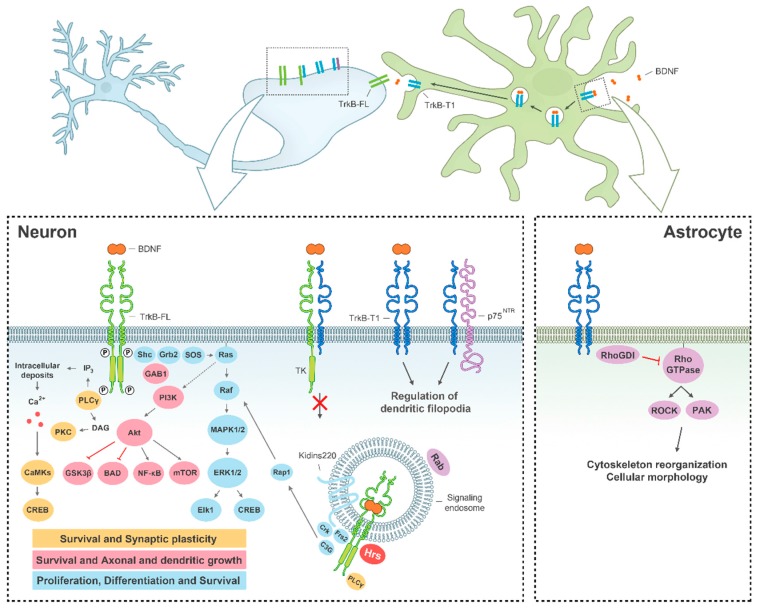
Function of BDNF/TrkB signalling in the CNS. BDNF binding to TrkB-FL in neurons (left diagram) induces receptor homodimerization and activation, triggering three main signalling pathways: MAPK/ERK (blue), PI3K (pink) and PLCγ (yellow), which regulate several processes central to neuronal function. The ligand-receptor complex can internalize and continue functioning in signalling endosomes. Alternatively, TrkB-T1 can form heterodimers with TrkB-FL and block its transduction cascades. TrkB-T1 is also involved in the regulation of local BDNF concentration (upper diagram) and cell morphology, both in neurons and astrocytes (respectively, left and right diagrams). P, phosphorylation sites important for receptor activation.

Along development and adult life BDNF can bind its high-affinity receptor TrkB, a transmembrane protein that mediates most of its biological functions, or the low-affinity receptor p75^NTR^, implicated in neurite growth and apoptosis. Four TrkB isoforms are expressed in human brain: the full-length receptor (TrkB-FL), two truncated isoforms TrkB-T1 and TrkB-Shc, which lack the tyrosine kinase domain, and TrkB-T-TK, having a non-functional catalytic domain [4]. Binding of BDNF to TrkB-FL induces the receptor dimerization and activation, and results in the recruitment of proteins that trigger three main signal transduction cascades which are widely interconnected (Figure 1) [5]. Phosphorylation of the Tyr515 residue (according to the TrkB-FL rat sequence) allows docking of Shc (Src-homology 2-domain containing adaptor protein) to the receptor. This is the initial step for activation of the PI3K/Akt cascade that controls the activity of several proteins essential for neuronal survival, such as BAD (Bcl-2 antagonist of cell death) or GSK-3β (Glycogen Synthase Kinase 3 β) [6]. Shc also triggers the action of the MAPK/ERK pathway, which promotes neuronal differentiation and survival through suppression of the proapoptotic protein BAD and activation of the transcription factor CREB (cAMP response-element binding protein) [7]. However, the prolonged activation of MAPKs requires binding of the adaptor protein FRS2 at the Tyr515 residue or action of TrkB-interacting protein Kidins220 (Kinase D interacting substrate of 220 kDa) to bring the downstream effectors in the vicinity of TrkB receptors [8]. The recruitment and activation of PLCγ (Phospholipase C γ) through phosphorylation of TrkB residue Tyr816 also promotes neuronal survival and is implicated in neurite outgrowth and synaptic plasticity [9]. The other major isoform expressed in the brain is TrkB-T1, which can be detected in neurons but is also present in glial cells. TrkB-T1 opposes TrkB-FL competing for BDNF [10] or forming inactive heterodimers in neurons [11]. However, the completely conserved short intracellular region and a brain expression pattern different from that of TrkB-FL suggest independent functions for TrkB-T1. Thus, the isoform present in glial cells can regulate the local concentration of BDNF [12] and is involved in cell morphology through interaction with Rho GDI (Rho GDP dissociation inhibitor 1) [13]. Interestingly, TrkB expression [14] and responsiveness to BDNF are developmentally regulated [15,16]. However, the postnatal decline of BDNF-induced TrkB-FL phosphorylation is not a consequence of the coincidental increase of TrkB-T1 expression or due to structural receptor modifications preventing BDNF binding [16]. Therefore, we still need to completely understand how TrkB mediates BDNF actions in the adult CNS. Finally, functional interactions established by TrkB isoforms and p75^NTR^ importantly contribute to the complexity of the neurotrophic response. These receptor systems are frequently co-expressed in the same cells and can form heteromeric complexes that change their affinity for mature neurotrophins [17].

The great diversity of biological functions regulated by the TrkB receptor in cells is also partially due to the compartmentalisation of receptor complexes. BDNF-bound TrkB receptors not only signal from the cell surface but also from endosomes that internalise BDNF/TrkB complexes together with activated components of their downstream effector pathways (Figure 1). Interestingly, depending on complex localisation, different signalling pathways can be activated. Additionally, early endosomes (also known as signalling endosomes) are dynamic structures that can modify their protein components in accordance to their cellular destinies: retrograde transport to neuronal soma to propagate the BDNF signal, recycling back to plasma membrane or lysosomal degradation [18]. The main responsible of conferring identity to endosomes is the family of Rab GTPases and, for example, late endosomes contain Rab7 and Rab9 while recycling endosomes are defined by the presence of Rab4 and Rab11 [19]. While TrkB-T1 is efficiently recycled back to the membrane through Rab4 endosomes, recycling of TrkB-FL is modulated by synaptic activity via a Rab11-dependent pathway [20,21].

## 3. Defective Expression and Stability of BDNF and TrkB in Neurological and Psychiatric Disorders

Given the central role played by BDNF/TrkB signalling in cell function, it is not surprising that changes in expression, traffic and/or stability of this neurotrophin and its high-affinity receptor are common mechanisms to many human pathologies. In general, aberrant neurotrophic signalling has been related to neurological and psychiatric disorders but also proliferative conditions, aging, obesity or hyperphagia related disorders, which have been revised elsewhere [22,23,24]. There is also an extensive literature about defective BDNF/TrkB signalling in cell and animal models of neurological and psychiatric disorders; therefore, we will prioritize here data obtained in preclinical models and human beings.

### 3.1. Molecular Mechanisms of BDNF/TrkB Dysfunction in Stroke

**Figure 2:**
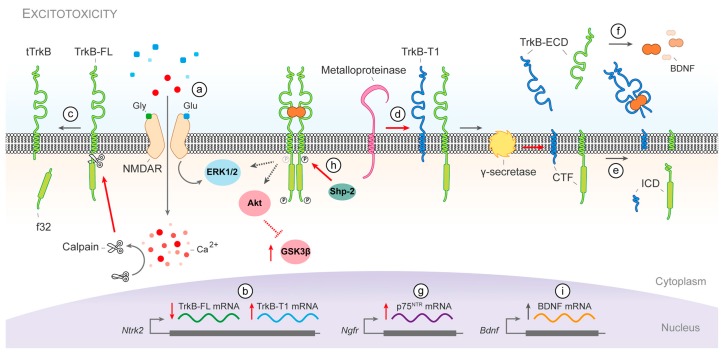
Dysfunction of BDNF/TrkB signalling during stroke. Excitotoxicity produced by overstimulation of the NMDARs (**a**) induces several mechanisms that dysregulate BDNF/TrkB signalling. The inversion of the physiological ratio of TrkB mRNA isoforms (**b**) and TrkB-FL cleavage by calpain (**c**) reduce the availability of the catalytic receptor and increase the dominant-negative forms. Furthermore, TrkB-FL and TrkB-T1 undergo a sequential cleavage first by metalloproteinases (**d**) and then by γ-secretases (**e**) that shed the receptor ectodomains, which then act as BDNF scavengers (**f**); BDNF can be further sequestered by increased expression of p75^NTR^ (**g**); Consequently, neurotrophic signalling is impaired, a situation aggravated even further by Shp-2 dephosphorylation of TrkB-FL at Tyr515 (**h**); Neurons in the peri-infarct area promote a survival response as a compensatory mechanism to brain damage and increase the expression of BDNF (**i**); CTF, C-terminal fragment; ECD, extracellular domain; f32, TrkB-FL calpain-fragment of 32 kDa; ICD, intracellular domain; tTrkB, calpain-truncated TrkB-FL.

Stroke is one of the leading diseases that affect the CNS. It is caused by decreased brain perfusion due to occlusion or haemorrhage of a blood vessel followed by deprivation of oxygen and nutrients in the deficiently irrigated tissue. This condition results in the formation of two differentiated brain areas: the infarct core, which is the region that suffers the most severe reduction in blood flow, surrounded by the penumbra, which is functionally impaired but remains metabolically active. However, after acute stroke, the penumbra frequently suffers processes of secondary neuronal death that cause the expansion of the infarct core over time. A central mechanism of neuronal death in the penumbra is excitotoxicity, produced by overstimulation of the *N*-methyl-d-aspartate type of glutamate receptors (NMDARs) (Figure 2a). Normal function of these receptors is very important to nervous system physiology because they are central to neurotransmission, neuronal survival and plasticity, and the processes of learning and memory.

In animal models of ischemic stroke, a permanent BDNF reduction has been observed in the infarct core, while rapid upregulation of neurotrophin expression lasting for several days was found in the penumbra [25,26]. However, BDNF has never been measured in the postmortem brains of stroke patients, although a slight increase in circulating neurotrophin levels observed after stroke could mirror brain levels [27]. Enhancement of BDNF production after stroke, mainly attributable to perilesional neurons but also to microglia [28], has been suggested as a brain compensatory mechanism to prevent excessive neuronal death [29]. However, several studies have concluded that BDNF is not involved in post-stroke functional recovery [30,31]. The most likely explanation for this outcome is the incapacity of BDNF to trigger appropriate neurotrophic signalling after stroke due to a pathological imbalance of TrkB receptor isoforms. In fact, levels of TrkB-FL diminish drastically in the infarcted core and penumbra area whereas those of TrkB-T1 are upregulated in human ischemic stroke [32] and animal models of ischemia [26,32]. These alterations are the consequence of three independent mechanisms induced by excitotoxicity. First, an inversion of the physiological ratio of the TrkB encoding mRNAs which favours the expression of the isoform TrkB-T1 over TrkB-FL (Figure 2b) [32,33]. The second mechanism is the calcium-dependent cleavage by calpain of TrkB-FL generating a truncated receptor similar to TrkB-T1 (Figure 2c), which might act as an additional dominant-negative receptor [32,33], and a cytosolic fragment of 32 kDa with the complete tyrosine kinase domain [32,34]. 

Finally, both isoforms undergo regulated intramembrane proteolysis (RIP) in neurons, shedding their ectodomains after the action of metalloproteinases (Figure 2d) followed by γ-secretases intramembrane processing of the remaining membrane-bound C-terminal fragments (Figure 2e). In a model of permanent ischemia in mice, RIP highly contributes to TrkB-T1 downregulation while it is a secondary mechanism for TrkB-FL, which is mainly processed by calpain [34]. Nevertheless, the secreted TrkB ectodomain, common to both isoforms contains the receptor domain important for ligand interaction and specificity and acts as a BDNF scavenger (Figure 2f) reducing even further the neurotrophic signalling [34]. In addition to TrkB-T1, calpain-truncated TrkB-FL and secreted TrkB ectodomains, BDNF can be also sequestered by p75^NTR^. The expression of this BDNF low-affinity receptor which mediates neuronal death is induced after cerebral ischemia (Figure 2g) [35]. All together, these mechanisms will severely inhibit binding of BDNF to its high-affinity receptor TrkB-FL.

Additionally, under excitotoxic conditions, TrkB-FL activity is also impaired by phosphatase Shp-2 (Src homology-2 domain-containing phosphatase-2) dephosphorylation of residue Tyr515 (Figure 2h) [36]. Consequently, downstream effectors of the neurotrophic signalling also suffer malfunction after stroke. Excitotoxicity induces an initial ERK1/2 activation in neurons which is followed by a gradual shut-off, traditionally attributed to the action of synaptic and extrasynaptic NMDARs respectively [37]. Impairment of TrkB-FL activity by Shp-2 action might also contribute to ERK1/2 inactivation in neurons. However, sustained ERK1/2 phosphorylation has been detected in the penumbra area after acute ischemic stroke in humans [38] and animal models [39], where it seems to contribute to brain injury [40]. Similarly, exposure to excitotoxic concentrations of glutamate inhibits PI3K/Akt and is followed by subsequent GSK-3β activation [41]. In the brain, levels of phosphorylated Akt increase within a few hours in the penumbra following ischemic damage but begin to decrease after 24 h [39], while GSK-3β activity varies depending on injury severity. Transient focal ischemia tends to activate GSK-3β and subsequently induce apoptotic cell death [42], whereas permanent cerebral ischemia rapidly suppresses it for 24 h after damage [43]. Finally, the transcription factor CREB is highly phosphorylated in the peri-infarct area in contrast to the core region. Since BDNF expression is CREB-regulated, this result supports the neurotrophin expression pattern observed after stroke (Figure 2i) [44]. Altogether, these results reflect an endogenous capacity of the brain to promote neurotrophic signalling after cerebral damage which is subverted by a truncation of the downstream signalling pathways, mainly at the receptor level, that blocks a proper neuronal survival response.

### 3.2. Molecular Mechanisms of BDNF/TrkB Dysfunction in Neurodegenerative Diseases

Excitotoxicity contributes to neuronal death in acute disorders other than stroke, as well as many chronic diseases of the CNS [45]. Therefore, it is not surprising that changes in BDNF/TrkB signalling pathways are similarly found in neurodegenerative diseases.

#### 3.2.1. Deficiency of BDNF/TrkB Signalling in Alzheimer’s Disease (AD)

**Figure 3:**
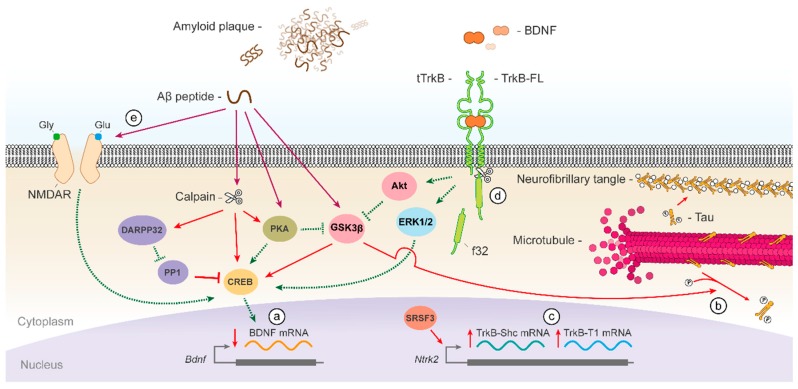
Dysfunction of BDNF/TrkB signalling in AD. Patients of AD show a decrease in BDNF levels in several brain areas due to a diminished gene expression (**a**). The consequent reduction in neurotrophic signalling results in the activation of GSK3β which contributes to tau hyperphosphorylation (**b**); Moreover, expression of truncated TrkB isoforms is favoured in AD brains by the action of transcription factor SRSF3 (**c**); Aβ peptide additionally promotes the activities of GSK3β and calpain, which cleaves TrkB-FL receptor near the receptor Shc docking site (**d**); Additionally, Aβ decreases CREB activity by several mechanisms including a reduction of NMDAR levels (**e**) and increased PP1 action. f32, TrkB-FL calpain fragment of 32 kDa; P, phosphorylation of tau residues; tTrkB, calpain-truncated TrkB-FL.

Neurotrophic signalling is severely impaired in AD. This progressive neurodegenerative disorder causes the most prevalent age-related dementia. It is characterized by the formation of senile plaques, which are extracellular deposits of misfolded amyloid β-peptide (Aβ), and intracellular neurofibrillary tangles composed of hyperphosphorylated tau protein (Figure 3). Patients with AD show a progressive decrease of synapses and, subsequently, of neurons mainly in the entorhinal cortex and hippocampus, circuitry essential for short-term memory [46,47,48]. Interestingly, levels of BDNF are reduced in those same brain areas in AD patients [49,50,51]. Regarding the presence of BDNF in serum, while we should be cautious due to some inconsistent results, a recent meta-analysis shows a significant decrease of neurotrophin levels in AD patients compared to healthy subjects [52]. The decrease of BDNF mRNA is an important mechanism of neurotrophin loss in AD brain (Figure 3a), observed in hippocampus [53], basal forebrain [54], and temporal [55] and parietal cortices [56]. Given its crucial role in neuronal survival, the lack of BDNF support will exacerbate the cognitive decline observed in AD. In fact, loss of proBDNF and BDNF occurs early in the disease course (before plaque deposition) and correlates with memory deficits [57,58], strongly suggesting the relevance of those changes for the synaptic loss and cell dysfunction underlying AD cognitive impairment. Moreover, BDNF induces rapid tau dephosphorylation in neuronal cells through TrkB activation and PI3K/Akt signalling, [59] and, therefore, this decrease in BDNF might also contribute to tau hyperphosphorylation (Figure 3b), a pathological hallmark of AD.

In addition to BDNF, a decrease in the TrkB-FL receptor has been found in postmortem AD brains, specifically in the nucleus basalis [60], and frontal [61,62] and temporal cortices [62]. Nevertheless, changes in TrkB-FL mRNA levels seem more controversial [63,64] suggesting that additional mechanisms participate in TrkB-FL downregulation. The decrease of TrkB-FL could be aggravated by the upregulation of truncated receptor isoforms TrkB-T1 and TrkB-Shc in AD brains (Figure 3c), taking place in frontal [61] and temporal lobes or the hippocampus [64,65]. However, again, some reports did not find any changes in TrkB-T1 levels in the cortex of patients [62].

Several mechanisms activated by the Aβ peptide, acting at different levels of the BDNF/TrkB signalling pathway, result in neurotrophic deficiency in AD. One prominent effector of this deathly Aβ activity is calpain which is overstimulated in postmortem AD brains [66]. Activation of this protease by Aβ in neuronal cultures induces a decrease of TrkB-FL [67] by cleavage near the receptor Shc docking site (Figure 3d) [68]. Similarly to stroke, this processing yields truncated TrkB-FL, which may act as a neurotrophin sink or dominant negative receptor, and the intracellular fragment with the complete tyrosine kinase domain. It has been previously suggested that the proteolytic fragments generated from receptor tyrosine kinases might regulate cell functions such as transcription or survival/apoptosis balance [69]. In addition, Aβ might be also inducing the upregulation of truncated TrkB isoforms in AD by transcriptional mechanisms [68,70]. Selective TrkB pre-mRNA splicing to produce TrkB-Shc transcripts is promoted by the splicing factor SRSF3 whose mRNAs levels are increased in AD and SHSY5Y cells treated with Αβ fibrils (Figure 3c) [70]. On the other hand, the decrease in BDNF levels is basically a consequence of aberrant transcription (Figure 3a), mainly due to CREB impairment in the hippocampus and frontal cortex of AD patients [71,72,73] by overlapping mechanisms. First, this transcription factor is proteolysed by calpain generating a truncated protein with reduced activity [72]. Additionally, protein kinase A (PKA), a major CREB regulator, is inactivated in the temporal cortex of AD patients [74,75] by Aβ action [76]. The inhibition of PKA signalling and, therefore, CREB function is attributed to calpain-dependent proteolysis of PKA RII subunits [75] and downregulation of PKA O-GlcNAcylation [77]. Aβ also decreases CREB activity by GSK3β overstimulation [78], which is produced by two mechanisms: decreased GSK3β inhibitory phosphorylation of Ser9 by PKA [79,80] and calpain proteolysis to yield a truncated GSK3β with augmented kinase activity [81,82]. Finally, Aβ also reduces CREB activity by decreasing NMDAR levels (Figure 3e) [83] and calpain-mediated cleavage of DARPP-32, a key inhibitor of PP1, phosphatase that regulates CREB dephosphorylation and inactivation [84].

Other important mechanisms contributing to the deficiency of BDNF/TrkB signalling in AD are the suppression of MAPK/ERK and PI3K/Akt pathways by sub-lethal concentrations of Aβ, without interference of TrkB-FL and PLCγ activation [85], and the disruption of BDNF-induced TrkB endocytosis. The exposure to Aβ oligomer can impair receptor endocytosis and downstream Akt activation through GSK3β-mediated dynamin 1 phosphorylation [86]. The oligomers also induce a deficit in BDNF-mediated TrkB retrograde trafficking [87] by disrupting ubiquitin [88] and calcium homeostasis [89]. Finally, mitochondrial dysfunction induced by Aβ is an early event in AD also conducting to deficits in BDNF axonal transport [90].

#### 3.2.2. Deficiency of BDNF/TrkB Signalling in Huntington’s Disease (HD)

This autosomal dominant neurodegenerative disorder is caused by a CAG expansion in the huntingtin (Htt) gene that results in elongation of the polyglutamine (polyQ) tract at the Htt N-terminus. Dysfunction and death of the medium-sized spiny neurons (MSNs) of the striatum is a primary pathological feature of this disease and main responsible for the motor, cognitive and psychiatric decline. It has been proposed that a deficiency of BDNF/TrkB signalling contributes to the selective vulnerability of MSNs in HD [91]. Reduced levels of striatal BDNF protein have been detected in HD patients at symptomatic disease stages [92] which are the result of decreased neurotrophin expression [93] and disrupted corticostriatal transportation [94]. It is important to consider that most BDNF in the striatum is synthesized and anterogradely delivered from cell bodies located in the cerebral cortex [95]. Remarkably, wild-type Htt is part of the motor complex responsible for anterograde and retrograde transport of BDNF-containing vesicles along microtubules [94]. In those complexes, Htt is associated with dynactin subunit p150^Glued^ via Htt-associated protein 1 (HAP1) [96,97] or directly with dynein [98]. The expanded polyQ tract of mutant Htt increases the association among complex components and leads to functional impairment and reduction of vesicle movement [94]. Thus, the tighter bond of mutant Htt to HAP1 in HD brain decreases this protein interaction with pro-BDNF [99]. An additional defect in HD brains affects tubulin acetylation and the recruitment of motor proteins to microtubules [100], which altogether leads to reduced neurotrophin release and transport.

The phosphorylation of wild-type Htt in Ser421 by Akt promotes the anterograde movement of vesicles [101], mediates IGF-1 neuroprotective effects in HD [102] and mitigates the toxicity of mutant Htt by increasing its proteasome-dependent turnover [103]. However, Akt is cleaved by caspase-3 into an inactive form in the postmortem brain of HD patients [102,104] supporting a prominent role for dysfunction of this survival pathway along disease progression. In addition to controlling neurotrophin transport, wild-type Htt also enhances *Bdnf* expression from promoter II [93] by sequestering repressor element-1 transcription factor/neuron-restrictive silencer factor (REST/NRSF) in the cytoplasm, suppressing its inhibitory transcriptional activity [105]. In contrast, mutant Htt is unable to retain REST/NRSF [105], leading to a reduction of BDNF mRNA levels in the cortex of HD patients [106]. Additionally, the expanded polyQ Htt generates a more repressive transcriptional environment for *Bdnf* by recruiting the methyl-CpG binding protein 2 (MeCP2) to promoter IV [107], also sequestering the transcriptional coactivator CREB binding protein (CBP) [108].

In addition to altering BDNF levels and transport, mutant Htt also leads to reduced neurotrophic support by affecting the availability of neurotrophin receptors. Thus, in HD patients there is an imbalance in the striatal expression of TrkB-FL mRNA with respect to p75^NTR^ and TrkB-Shc [106]. Consequently, high p75^NTR^ but decreased TrkB-FL protein levels are observed in the striatum from HD patients at late disease stages [109,110]. The upregulation of Sp1 observed in cellular and transgenic models of HD [111] could underlie the increased expression of p75^NTR^ since this gene is regulated by this transcription factor [112]. In addition to the transcriptional imbalance, mutant Htt can also alter binding of TrkB-FL-containing vesicles to microtubules and impair retrograde endosomal trafficking in striatal dendrites [113]. Furthermore, induction by mutant Htt of deficient Rab11 activity [114] could reduce TrkB cell surface availability since this GTPase is typically involved in TrkB-FL endosomal recycling [21,115]. Additionally, calpain is overactivated in the striatum of human HD tissue [116] and, therefore, it might too cleave the TrkB-FL receptor in this disease.

The deficiency in BDNF/TrkB downstream signalling observed in HD has been recently revealed to precede the defects in transport and expression of neurotrophin and receptors [117,118]. Thus, the synaptic dysfunction of MSNs early in HD is attributable to enhanced p75^NTR^ signalling through PTEN (phosphatase and tensin homolog) resulting in suppression of Akt signalling [117,119]. Likewise, striatal activation of TrkB-FL and ERK1/2 is attenuated at early disease stages when total receptor and ligand levels are still normal [118]. However, the characterization of kinase signalling in HD models is still controversial [120], highlighting the need to redefine the timeline of the deficits in neurotrophic effectors in order to develop therapies to treat involuntary movement in symptomatic HD patients.

#### 3.2.3. Deficiency of BDNF/TrkB Signalling in Parkinson’s Disease (PD)

The most common neurodegenerative movement disorder, PD is characterized by the progressive loss of dopaminergic neurons in the substantia nigra pars compacta (SNpc) along with defective intracellular accumulation of α-synuclein inclusions, the so-called Lewy bodies and Lewy neurites. Postmortem studies of PD patients reveal a reduction of BDNF mRNA and protein in the vulnerable region SNpc [121,122] and also the striatum [123], which receives neurotrophic support from the SN [95]. In contrast, levels of TrkB-FL mRNA are normal in surviving SNpc neurons of PD brains [124] while only minimal regional changes are observed in protein levels [125]. However, there is an important shift in the subcellular distribution of TrkB-FL and TrkB-T1 in PD SNpc and striatum [125], being prominent a decrease of the catalytic receptor isoform in dendrites indicative of impaired synaptic function. Nonetheless, the deficiency in BDNF/TrkB survival signalling increases the susceptibility of SN dopaminergic neurons to cytotoxic injury [126,127] and might contribute to PD development. Actually, inhibition of BDNF expression or TrkB insufficiency cause selective loss of SNpc dopaminergic neurons [128,129,130] and exacerbate motor dysfunction in aged animals [131]. A possible feedback mechanism contributing to this selective effect might be the increase in α-synuclein levels produced in response to a deficit of TrkB-FL [128].

Nevertheless, it is generally assumed that the impairment of neurotrophic signalling in PD is the consequence of the toxicity and prion-like propagation of misfolded α-synuclein [132]. Interestingly, aggregates of α-synuclein do not cause a generalized defect in axonal transport but specifically impair that of TrkB-FL-containing late endosomes [133]. Since late endosomes control TrkB receptor retrograde delivery [134], this observation could explain the shift in receptor subcellular location found in PD [125] and mentioned before. Furthermore, α-synuclein overexpression also alters pathways required for neurotrophic signalling. Thus, elevated levels of α-synuclein increase the activity of the Akt inhibitors phosphatase PP2A [135] and RTP801 [136] while Akt phosphorylation is significantly diminished in dopaminergic SN neurons of PD patients [137]. Attenuation of Akt phosphorylation leads to GSK3β activation in the presence of α-synuclein aggregates [138]. Additionally, human postmortem tissues corresponding to different Lewy body diseases exhibit granular cytoplasmic aggregates of activated ERK in the SN, probably formed early along the disease course, that may affect the accessibility to downstream targets and regulatory phosphatases [139]. α-synuclein also contributes to downregulation of neurotrophin transcription by suppression of Elk-1 activity [140] and competition in nuclei with CREB for binding to CREs in promoter regions [141]. 

Finally, mitochondrial dysfunction alters calcium homeostasis in PD leading to the overactivation of calpain [142] which then, as before, may act on different substrates important to neurotrophic signalling. In addition, calpain also processes α-synuclein [143], a truncation that leads to formation of high-molecular weight aggregates [144]. Thus, calpain activation has been suggested to participate in disease-linked α-synuclein aggregation in PD as well as other α-synucleopathies [144,145].

### 3.3. Molecular Mechanisms of BDNF/TrkB Dysfunction in Other Pathologies

Malfunction of BDNF/TrkB also plays a role in the pathophysiology of psychiatric disorders although the available evidence is still limited. Among them, one of the better characterised diseases is depression. Several lines of evidence indicate that it may be associated with the inability of neuronal systems to exhibit adaptive plasticity, and highlight the reduction in neurotrophic signalling as one central disease mechanism (reviewed in reference [146]). Stress, considered a major risk factor for depression, decreases BDNF and its downstream signalling in the hippocampus and cerebral cortex [147]. Studies with antidepressants also support the neurotrophic hypothesis of depression, since chronic treatment with them increases blood BDNF levels in patients [148,149]. Moreover, antidepressants also upregulate the expression of TrkB mRNA [150] and induce a rapid activation of this receptor and the PLCγ mediated signalling [151]. Simultaneously, it has been shown that the therapeutic effects of antidepressants require the action of the BDNF/TrkB pathway [152] and, furthermore, that centrally administered BDNF provides a similar effect to antidepressants in animal models of depression [153]. Interestingly, the reduction of BDNF and the two major TrkB isoforms is also evident in the postmortem brain of suicide victims [154,155], generally having a high incidence of previous major depression. This decrease in TrkB isoforms has been associated with a failure of the E3 ligase c-Cbl, a protein involved in TrkB-FL stabilization by ubiquitination [156], and an increase of Hsa-miR-185*, a microRNA responsible for the regulation of TrkB-T1 expression [157].

Schizophrenia patients suffer impairments in perception, cognition and motivation that reflect, at least in part, deficits in dendritic spines [158]. An essential event in the pathogenesis of schizophrenic psychoses is aberrant expression of neurotrophic factors, proposed to be responsible for disturbed neural development and plasticity. Thus, several studies have shown decreased circulating BDNF levels in individuals with schizophrenia [159]. Simultaneously, alterations in BDNF protein [160,161] and total mRNA have been observed in postmortem prefrontal cortex from patients [161,162]. Aberrant DNA methylation might be involved in this altered BDNF regulation, as reduced binding of GADD45b (a growth arrest and DNA-damage-inducible β protein) to one of *Bdnf* promoters has been observed in psychotic subjects [163]. Additionally, decreased levels of TrkB mRNA [162,164] and protein, together with reduced activity of TrkB downstream effectors Akt and ERK1/2 [165,166], have been reported in the prefrontal cortex of schizophrenia patients. By contrast, expression of the truncated isoforms TrkB-Shc and TrkB-T1 undergoes an increase in the brain of schizophrenic subjects [167].

Other pathologies related to decreased BDNF/TrkB signalling are neurodegenerative diseases of the retina such as glaucoma, age-related macular degeneration, diabetic retinopathy or retinitis pigmentosa. For glaucoma, death of retinal ganglion cells (RGCs) resulting in optic nerve damage and irreversible blindness can be explained by a lack of neurotrophic support [168]. The most important risk factor for glaucoma is intraocular pressure [169]. Interestingly, an acute elevation of intraocular pressure in experimental glaucoma leads to an obstruction of BDNF retrograde axonal transport from central target cells to the RGC soma [170] and accumulation of TrkB in the optic nerve head [171]. The subsequent defect in neurotrophic signalling leads to RGCs apoptotic death. Several studies have shown that BDNF transiently delays RGC death in glaucoma [172,173]. Therefore, besides treatments directed to decrease intraocular pressure, neurotrophic factors are currently considered as having great potential in glaucoma therapy (recently reviewed by [174]).

Finally, excessive activation of TrkB-FL has been unveiled as a molecular mechanism underlying the induction of epilepsy [175], which is broadly characterized by aberrant neuronal excitability. To promote epilepsy, this abnormal TrkB signalling requires the action of the PLCγ pathway [176]. However, chronic seizures can alter neuronal and glial expression of glutamate receptors and uptake transporters, which then trigger excitotoxicity and cause permanent neurological damage [177]. Accordingly, in vitro models of recurrent epileptic seizures lead to the characteristic imbalance of the BDNF receptors, with a decrease of TrkB-FL produced by calpain cleavage and upregulation ofTrkB-T1 and p75^NTR^ levels [178,179]. It will be interesting to investigate if the intracellular TrkB-FL calpain fragment maintains PLCγ interaction, as described for similar TrkA fragments [180], and further exacerbates epileptogenesis.

## 4. Restoration of the BDNF/TrkB Pathway Requires Combined Targeting of BDNF and TrkB

The disorders and pathological conditions induced or promoted by aberrant BDNF/TrkB signalling could be potentially treated by fine-tuned activation (e.g., stroke, neurodegenerative diseases) or suppression (e.g., epilepsy, cancer) of this pathway. Particularly, in the case of neurological disorders, the recovery of neurotrophic signalling could be not only neuroprotective but also promote adult neurogenesis (reviewed in [181]) or synaptic plasticity and growth [182], which are altered in many of these diseases.

Different strategies directed to increase the availability of BDNF have been evaluated. Several research groups have shown neuroprotective effects in disease models induced by treatment with recombinant BDNF. However, administration of this neurotrophin did not exhibit the expected results in clinical trials [1,2] mostly due to poor BDNF transfer across the BBB and tissue diffusion, short serum half-life and important side effects (diarrhoea, paraesthesias, sleep disturbance or injection site reactions) [3]. To improve BDNF delivery, other approaches currently under consideration are nanoparticle-mediated transport, gene therapy with BDNF-encoding viral vectors or transplantation of BDNF-releasing cells (reviewed in reference [183]). It is worth mentioning that, compared to native BDNF, a nanoparticle formulation of BDNF significantly decreases the loss of brain tissue in mice when administered up to 6 h after stroke onset. More delayed treatment (12 h) still improves memory/cognition and reduces post-stroke depression but has no effect on infarct size [184]. So, even in situations of compromised BBB integrity such as stroke, BDNF nanoparticles are still more efficient than native BDNF improving neuropathological and neurobehavioral outcomes. Nevertheless, caution should be taken before systemic BDNF administration since the neurotrophin might interfere with activity-dependent neuronal plasticity, learning and memory, or even initiate epileptic activity [185]. Different laboratories are working in strategies to specifically reach the damaged areas or nearby tissue. Thus, a theranostic nanocarrier that specifically targets the peri-infarct tissue in cerebral ischemia has been developed [186]. This nano-platform contains imaging probes for visualization by conventional imaging techniques, a therapeutic agent for treatment and an antibody that directs to the desired region.

An interesting alternative to BDNF administration is the enhancement of endogenous neurotrophin production. For example, physical exercise evokes a significant increase of BDNF levels in rat hippocampus and cerebral cortex [187] which is mediated by brain uptake of circulating insulin-like growth factor I (IGF-I) [188]. Several clinical trials focused in the elderly and patients of stroke and neurodegenerative diseases, have reported the induction by exercise of a cognitive improvement together with an increase in BDNF levels (reviewed in reference [189]). A slow increase in the synthesis of BDNF has been similarly reported in response to chronic treatment with monoamine-based antidepressants, not only in rat brain [150] but also in patients of major depressive disorder (MDD) [190]. More recently, it has been discovered that the NMDAR antagonist, ketamine, produces faster (within hours) antidepressant responses in MDD patients resistant to conventional treatments, concurrent with an increase in the number and function of synaptic connections and enhancement of BDNF expression [191]. A novel study has discovered that one specific enantiomer of a ketamine metabolite can exert rapid and sustained antidepressant actions in mice, which are independent of NMDAR inhibition but require AMPAR activation, while lacks ketamine unwanted side effects [192]. Thus, both gradually and rapid acting antidepressants reverse the significant decrease of BDNF levels characteristic of MDD patients [193] and promote TrkB signalling, synaptic plasticity and neuronal excitability. Finally, another promising approach has been the development of a small-size orally active molecule (PYM50028) able to increase levels of GDNF and BDNF in the striatum of MPTP-lesioned mice, considered as a good candidate for neuroprotection and neurorepair in PD [194]. An anticipated limitation of strategies aimed to enhance endogenous BDNF expression might derive from recent discoveries showing that aging triggers a repressive chromatin state in mice hippocampus at *Bdnf* promoters, which do not respond to synaptic activity and may contribute to cognitive decline [195]. Since most of the acute and chronic pathologies presenting decreased neurotrophin availability affect to the elderly, these results may imply that the efficiency of therapies aimed to increase endogenous BDNF will be compromised. However, an additional observation of these experiments is that the pharmacological prevention of age-associated cholesterol loss rescues BDNF expression and cognitive deficits in old mice [195]. These results are highly relevant and could facilitate the design of future therapies aimed to enhance BDNF expression in humans, where a decrease in cholesterol content has been also reported in normal aging brain and AD patients [196,197].

Another strategy has consisted in the development of small-size TrkB agonists alternative to BDNF, or BDNF mimetics, such as 7,8-dihydroxyflavone (7,8-DHF), an stable molecule able to efficiently cross the BBB after oral administration [198]. 7,8-DHF acts as a selective and efficient TrkB agonist and presents neuroprotective effects in excitotoxic processes induced in vitro [199] or using in vivo models of brain ischemia [198], AD [200], ALS [201] or PD [198], among others. Thus, flavonoid-based TrkB agonists are currently considered as very promising compounds to treat stroke and neurodegenerative diseases. An alternative to TrkB agonists for neuroprotection is transactivation of Trk receptors by ligands of G protein-coupled receptors (GPCRs), dopamine or glucocorticoids (reviewed in reference [202]). Brain TrkB transactivation is also achieved by antidepressant drugs in adult mice via unknown mechanisms [16] that result in specific phosphorylation of Tyr816, PLCγ activation and CREB phosphorylation [151], processes that are independent of monoamine transporter inhibition or BDNF action [203].

In any case, the efficiency of treatments enhancing or mimicking BDNF actions, or those directed to TrkB transactivation, could be dramatically limited if the receptor stability and function were aberrant, as is frequently the case in neurological and psychiatric disorders. Therefore, we necessarily need to devise therapeutic compounds that recover TrkB receptor and downstream signalling to be used in combination with drugs acting upstream. This is particularly important in the case of diseases with an excitotoxic component due to calpain and metalloproteinase/γ-secretase activation. As mentioned, isoforms TrkB-FL and TrkB-T1 are RIP substrates and release a receptor ectodomain that acts as a BDNF scavenger and significantly alters BDNF/TrkB signalling [34]. Additionally, TrkB-FL is also a substrate of calpain that produces a truncated receptor form suggested to act as a dominant negative protein in several pathologies [32,68,178]. Several studies have already shown a neuroprotective effect for the recovery of TrkB isoforms relative levels. Thus, combined interference of TrkB-T1 overexpression and increased TrkB-FL synthesis in a cellular model of excitotoxicity allows for recovering a TrkB-FL/TrkB-T1 balance and protects neurons from excitotoxic death [32]. Likewise, overexpression of TrkB-FL in a mouse model of AD alleviates spatial memory impairment while TrkB-T1 overexpression further exacerbates these alterations [67]. Finally, in an animal model of Down syndrome, where mice have normal TrkB-FL levels but upregulated TrkB-T1, restoration of physiological TrkB-T1 expression rescues cortical and hippocampal neurons from death, corrects resting Ca^2+^ levels and restores BDNF-induced intracellular signalling [204].

One of the more innovative ideas for the modulation of BDNF/TrkB downstream signalling pathways is shuttle-mediated drug delivery by conjugation of the therapeutic molecules to cell-penetrating peptides (CPPs) capable to cross the BBB and the cell membrane (reviewed by [205]). Thus, a CPP has been recently developed that contains a short HIV-1 Tat sequence, a favourite carrier peptide, and the dynamin 1 sequence phosphorylated by GSK3β. Specific inhibition by this CPP of GSK3β-induced dynamin 1 phosphorylation in neuronal and mouse models of AD rescues impaired BDNF-dependent TrkB endocytosis and Akt activation [86]. Likewise, Tat peptides linked to specific sequences cleaved by calpain have proven to be effective to prevent action of this protease on the corresponding substrates in models of excitotoxicity in vitro or in vivo. For example, this has been demonstrated for STEP (striatal-enriched protein tyrosin phosphatase) [206], an important regulator of synaptic signalling proteins or the NMDAR, or the metabotropic glutamate receptor 1 [207]. Similarly, a CPP containing a short Kidins220 sequence enclosing the major calpain site identified in this protein improves neuronal viability by preserving the activity of ERK1/2 and CREB after an excitotoxic insult [208]. All this evidence shows that maintenance of the survival pathways truncated by the pathological action of calpain is an effective neuroprotective strategy. Moreover, these results paves the way for the design and development of CPPs targeting other key neurotrophic effectors impaired in neurological diseases like PKA, CREB or even the TrkB receptors. In fact, TrkB-FL has already been considered a therapeutic target for epilepsy prevention. Contrary to most neurological disorders, here it is necessary to counteract the epileptogenesis overactivation of TrkB-FL [175]. Since PLCγ signalling has a prominent role in this pathological TrkB action, a Tat peptide has been designed able to uncouple these two proteins [176]. Treatment with such peptide prevents epilepsy and anxiety-like disorder without altering the neuroprotective effects of endogenous TrkB signalling.

## 5. Conclusions

In this article, we have reviewed evidence demonstrating that dysregulation of neurotrophic signalling is common to most neurological disorders, including stroke and neurodegenerative diseases, and that alterations are produced at different levels of this route. Altogether, the presented data highlight the importance of this key pathway for the treatment of neurological disorders and show the necessity of approaching the development of therapies in a more integral way. The enhancement of the BDNF/TrkB signalling pathways will certainly require the combination of BDNF targets with those addressing the aberrant expression and function of TrkB receptors and downstream effectors.

## Abbreviations

7,8-DHF	7,8-Dihydroxyflavone
Aβ	Amyloid β-Peptide
AD	Alzheimer’s Disease
ALS	Amyotrophic Lateral Sclerosis
BAD	Bcl-2 Antagonist of Cell Death
BBB	Blood–Brain Barrier
Bcl-2	B-Cell Lymphoma 2
BDNF	Brain-Derived Neurotrophic Factor
CBP	CREB Binding Protein
CREB	cAMP Response-Element Binding Protein
GPCRs	G Protein-Coupled Receptors
HAP1	Huntingtin-Associated Protein 1
HD	Huntington’s Disease
IGF-1	Insulin-like Growth Factor I
MDD	Major Depressive Disorder
MeCP2	Methyl-CpG Binding Protein 2
MSNs	Medium-sized Spiny Neurons
NMDARs	*N*-methyl-d-aspartate Type of Glutamate Receptors
p75^NTR^	p75 Neurotrophin Receptor
PD	Parkinson’s Disease
PKA	Protein Kinase A
polyQ	Polyglutamine
PTEN	Phosphatase and Tensin Homolog
REST/NRSF	Repressor Element-1 Transcription Factor/Neuron-Restrictive Silencer Factor
RGC	Retinal Ganglion Cell
RhoGDI	Rho GDP Dissociation Inhibitor 1
RIP	Regulated Intramembrane Proteolysis
Shc	Src-Homology 2-Domain Containing Adaptor Protein
Shp-2	Src Homology-2 Domain-Containing Phosphatase-2
SN	Substantia Nigra
SNpc	Substantia Nigra pars compacta
STEP	Striatal-Enriched Protein Tyrosin Phosphatase
Trk	Tropomyosin-Related Kinase
TrkB-FL	Full-Length TrkB
